# Surgical treatment of bicuspid aortic valve disease

**DOI:** 10.1186/1749-8090-8-S1-P7

**Published:** 2013-09-11

**Authors:** Ivan Kravchenko, VI Kravchenko, LL Sytar, OV Pantas, SO Dykuha, MY Atamanyuk, GV Knyshov

**Affiliations:** 1 Department of Aortic Surgery, M. Amosov National Institute of CVS NAMS Ukraine, Kyiv, Ukraine; 2 Department of Congenital Heart Liason, M. Amosov National Institute of CVS NAMS Ukraine, Kyiv, Ukraine; 3 M. Amosov National Institute of CVS NAMS Ukraine, Kyiv, Ukraine

## Background

Congenital bicuspid aortic valve (BAV) – one of the most frequent cardiovascular lesions, with prevalence up to 2.4%. About a half of them need the surgical treatment throughout life.

## Methods

1217 patients with BAV were treated in the Institute during 2003−2012. There were 902 (74.1%) males. Patients age ranged from 3 days to 76 years, mean 50.2±9,8 years. Aneurysm of ascending aorta took place in 380 (31.2%) pts, 39 (10,3%) of them admitted with dissection. We use: balloon valvuloplasty in 82 (8.1%) patients. Different operative interventions were fulfilled to the rest :1135 (93,3%) pts: open aortic valvotomy − 64 (5,3%), aortic valve replacement (AVR) – 691 (56.8%), AVR with ascending aorta banding – in 83 (6,8%), Robicsek operation – 121 (10.0%), Bentall’s operation – 162 (13.3%), Wheat’s operation – 9 (0.7%), supracoronary aortic grafting with AV resurpension – 3 (0.2%), David’s operation – 2 (0.2%); 51 (4.2%) patients with BAV treated of coarctation of the aorta earlier.

## Results

Total 30-day mortality composed 2.3% (26 pts). Death causes: acute heart failure – in 13 (1,1%); respiratory insufficiency – in 4 (0.3%), bleeding – in 2 (0.2%), multiorgan failure – in 4 (0,3%), cerebral injury – in 3 (0.2%). Remote results were studied in 1110 (93.7%) discharged patients in term 6 months – 9 years, mean 3,6±1,2. In mostly – 84.4% and 10.9% it was good and satisfactory. Unsatisfactory in 23 (2.0%), died in remote terms 27 (2,4%) pts. Redo operation were performed in 14 (2.1%) pts after AVR in a reason of aneurysm forming.

## Conclusion

Bicuspid aortic valve disease characterized by injury of aortic valve and ascending aorta. Aneurysm forming were observed in 380 (31.2%) patients with BAV, among them in 39 (10.3%) with dissection. Redo operations in a reason of aneurysm forming needs 2.1% in the elective AVR group. Operative treatment of patients with BAV permitted to obtain good and satisfactory remote results in 84.4% and 10.9% cases.

